# From Report to State Medical Society

**Published:** 1854-11

**Authors:** D. L. M’Gugin


### EXTRACT FROM A REPORT TO THE IOWA STATE MEDICAL SOCIETY.

BY PROF. D. L. M'GUGIN.

*A remarkable case of prolonged vital contractility in the heart of a fish.*—In May, of this year, a fish, weighing a little over half a pound it is supposed, was caught in the Mississippi, sold at the wharf and purchased by one of our household, with others, for the table. At the time of the purchase the fish betrayed no signs of life, a small cat-fish, bought at the same time, from the same fish-monger, did give repeated manifestations of vitality. It was brought to the house, a distance of a fourth of a mile from the place where purchased, and during this time it evinced no movement betokening life. A youth in our employ, commenced the work of removing the scales, which when done, the small fish above alluded to was decapitated, and aftewards disemboweled. He discovered above the viscera removed, a small substance pulsating after the manner of the heart, and as he had paid some attention to Anatomy, having dissected during two or three of the past winters, he soon decided it to be the heart. It continued to pulsate, he supposed, from the first discovery of a movement for about 12 minutes, during which time it was wholly separated from the fish, or any part of it. He then called my attention to it, and it was found to be the heart, and the *systolic* and *diastalic* movements regularly and fully performed. It was watched for half an hour, during which it continued to contract and expand as regularly as at first notice. Being called away in the meantime, I requested Mr. Robert Adams, son of Dr. Adams, deceased, of Pennsylvania, to observe it and mark the time when its movements ceased. In one hour and a half I returned, and to my astonishment, I found it still dilating and contracting, but at somewhat longer intervals. It still continued its movements, each becoming more and more feeble, until at the expiration of two hours and forty minutes after its separation and removal from the fish, it ceased its action. Prior to this time some ten minutes, Dr. Haines, of this city, called, and upon mentioning the case to him, was invited to see it. Although very feebly, and at long intervals, it continued its movement, yet it was plainly perceptible to us all, and by Dr. Haines in particular, who assisted in weighing it and found it to be four grains only.—The heart is now in my possession.

Here is furnished a strong corroborating proof of the postulate that "contractility is a property of muscular tissue, and inherent in it, and that the agency of the nervous system upon it, is merely to call it into active operation." In this case there was an entire separation from the nervous center and ganglia, nor was there any stimulus derived from the blood. The nutrition, which had been supplied to it and retained, appears to have been the only source of stimuli which, acting upon its special nerves, perpetuated its contractility. It is quite reasonable then, to suppose that stimuli act upon the nerves, and the nerves in turn, through their filamentous distribution, furnish to muscular fibres their capacity for contractility.

